# Trisodium citrate, Na_3_(C_6_H_5_O_7_)

**DOI:** 10.1107/S2056989016007453

**Published:** 2016-05-10

**Authors:** Alagappa Rammohan, James A. Kaduk

**Affiliations:** a Atlantic International University, Honolulu, HI, USA; b Illinois Institute of Technology, Chicago, IL, USA

**Keywords:** crystal structure, powder diffraction, density functional theory, sodium citrate

## Abstract

The crystal structure of anhydrous tris­odium citrate, Na_3_(C_6_H_5_O_7_), has been solved and refined using synchrotron X-ray powder diffraction data, and optimized using density functional theory (DFT). There are two independent five-coordinate Na^+^ and one six-coordinate Na^+^ cations in the asymmetric unit. The [NaO_5_] and [NaO_6_] polyhedra share edges and corners to form a three-dimensional framework. There are channels parallel to the *a* and *b* axes in which the remainder of the citrate anions reside. The only hydrogen bonds are an intra­molecular one between the hy­droxy group and one of the terminal carboxyl­ate O atoms and an intermolecular one between a methylene group and the hydroxyl O atom.

## Abstract 2

The crystal structure of anhydrous tris­odium citrate has been solved and refined using synchrotron X-ray powder diffraction data, and optimized using density functional techniques. The five-, six-, and five-coordinate Na polyhedra share edges and corners to form a three-dimensional framework.

## Chemical context   

In the course of a systematic study of the crystal structures of Group 1 (alkali metal) citrate salts to understand the anion’s conformational flexibility, ionization, coordination tendencies, and hydrogen bonding, we have determined several new crystal structures. Most of the new structures were solved using powder diffraction data (laboratory and/or synchrotron), but single crystals were used where available. The general trends and conclusions about the 16 new compounds and 12 previously characterized structures are being reported separately (Rammohan & Kaduk, 2016*a* ▸). Two of the new structures containing multiple Group 1 cations) – NaKHC_6_H_5_O_7_ and NaK_2_C_6_H_5_O_7_ – have been published recently (Rammohan & Kaduk, 2016*b* ▸,*c* ▸).
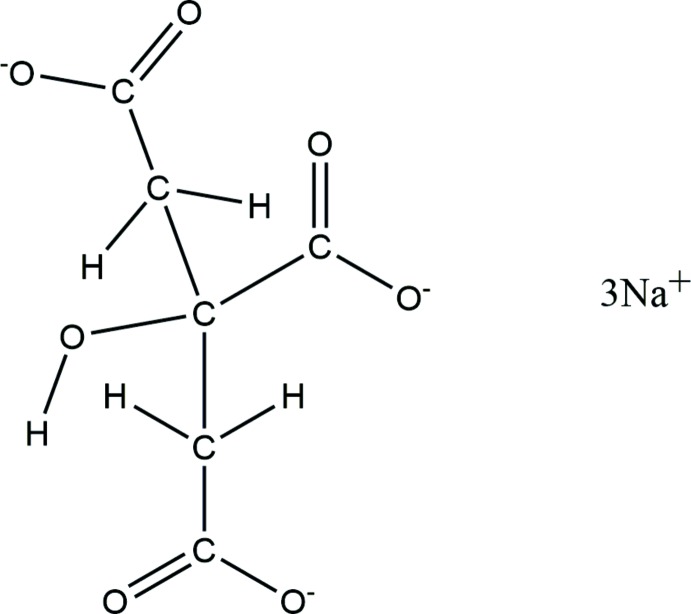


## Structural commentary   

**Figure 1:**
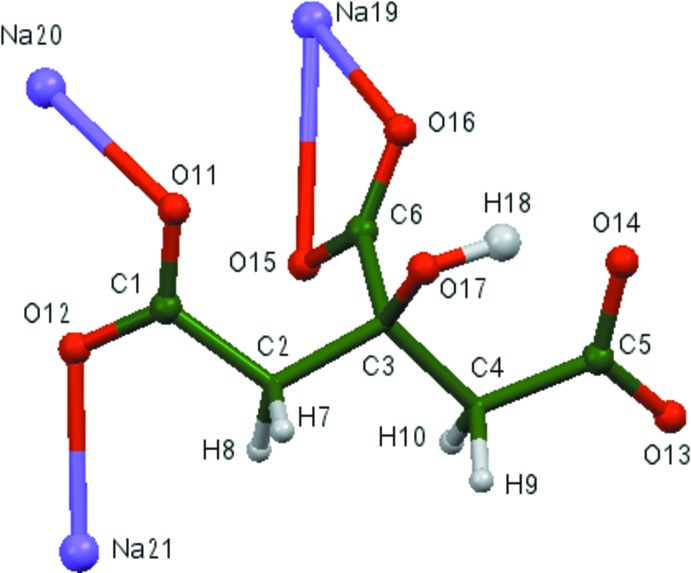
The asymmetric unit, showing the atom numbering. The atoms are represented by 50% probability spheroids.

**Figure 2:**
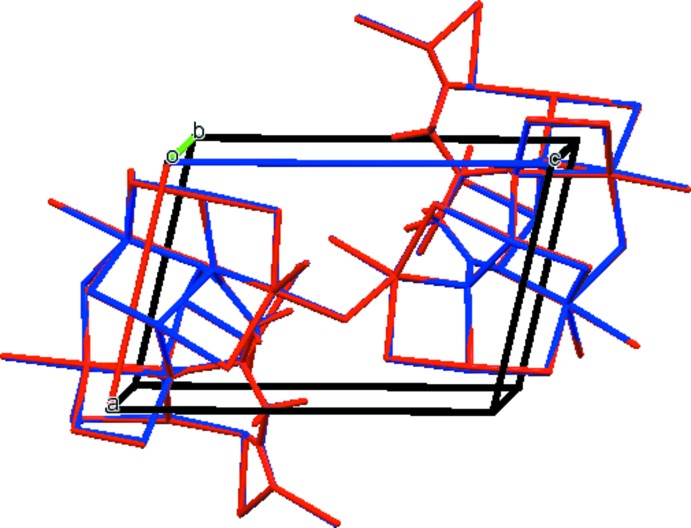
Comparison of the refined and optimized structures of tris­odium citrate. The refined structure is in red, and the DFT-optimized structure is in blue.

The asymmetric unit of the title compound is shown in Fig. 1 ▸. The root-mean-square deviation of the non-hydrogen atoms in the Rietveld refined and the optimized structure using density functional theory (DFT) is only 0.057 Å. The maximum deviation is 0.103 Å, at Na19. The excellent agreement between the two structures (Fig. 2 ▸) is strong evidence that the experimental structure is correct (van de Streek & Neumann, 2014 ▸). This discussion uses the DFT-optimized structure. All of the bond lengths, bond angles, and torsion angles fall within the normal ranges indicated by a *Mercury Mogul* geometry check (Macrae *et al.*, 2008 ▸). The hydroxyl group bridges atoms Na20 and Na21. The citrate anion occurs in the *trans*,*trans*-conformation (about C2—C3 and C3—C4), which is one of the two low-energy conformations of an isolated citrate. The central carboxyl­ate group and the hydroxyl group occur in the normal planar arrangement. The central carboxyl­ate group C6–O15–O16 chelates to Na19, and the terminal carboxyl­ate C5–O13–O14 chelates to Na21. The citrate chelates to Na20 through the hydroxyl group O17 and the terminal carboxyl­ate C1–O11–O12, and to a second Na19 through the terminal carboxyl­ate oxygen atom O14 and the central carboxyl­ate oxygen atom O16. Na19 is five-coordinate (irregular) with a bond-valence sum of 1.08. Na20 is six-coordinate (distorted octa­hedral) with a bond-valence sum of 1.14. Na21 is five-coordinate (trigonal–bipyramidal) with a bond-valence sum of 1.01. The metal–oxygen bonding is ionic, based on the cation charges and Mulliken overlap populations.

## Supra­molecular features   

**Figure 3:**
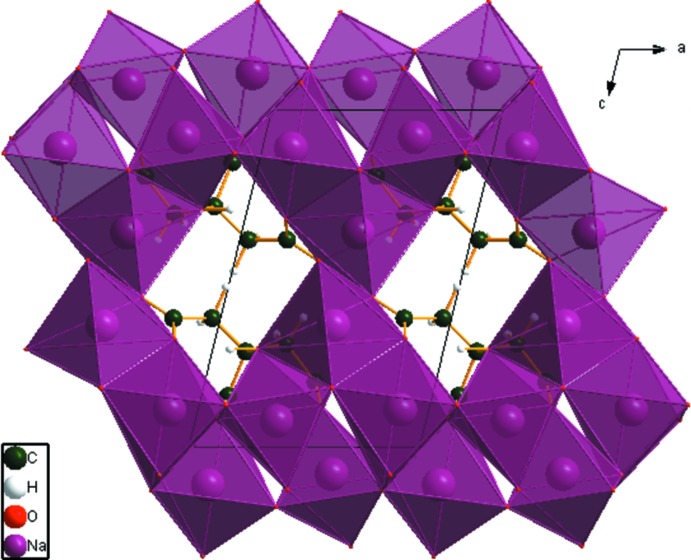
Crystal structure of Na_3_(C_6_H_5_O_7_), viewed down the *b* axis.

There are two independent five-coordinate and one six-coordinate Na^+^ cations in the asymmetric unit. The [NaO_5_] and [NaO_6_] polyhedra share edges and corners to form a three-dimensional framework (Fig. 3 ▸). There are channels parallel to the *a* and *b* axes in which the remainder of the citrate anions reside. The only hydrogen bond is an intra­molecular O17–H18⋯O14 one between the hy­droxy group and one of the terminal carboxyl­ate O atoms (Table 1 ▸). One inter­molecular C—H⋯O hydrogen bond also apparently contributes to the crystal packing.

## Database survey   

Details of the comprehensive literature search for citrate structures are presented in Rammohan & Kaduk (2016*a* ▸). A reduced cell in the Cambridge Structural Database (Groom *et al.*, 2016 ▸) search (increasing the default tolerance from 1.5 to 2.0%) yielded 19 hits, but limiting the chemistry to C, H, Na, and O only resulted in no hits. The powder pattern matched no entry in the Powder Diffraction File (ICDD, 2015 ▸).

## Synthesis and crystallization   

The sample was purchased from Sigma–Aldrich (lot #119K0107V) as anhydrous Na_3_(C_6_H_5_O_7_). A laboratory powder pattern confirmed its phase purity. In the one year between this measurement and the measurement of the synchrotron pattern, the sample had partially hydrated to contain Na_3_(C_6_H_5_O_7_)(H_2_O)_2_ (UMOGAE; Fischer & Palladino, 2003 ▸).

## Refinement details   

**Figure 4:**
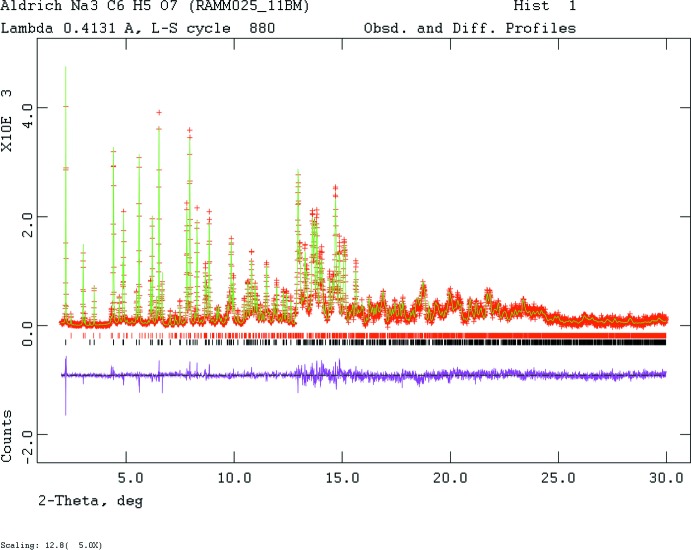
Rietveld plot for the refinement of Na_3_(C_6_H_5_O_7_). The red crosses represent the observed data points, and the green line is the calculated pattern. The magenta curve is the difference pattern, plotted at the same scale as the other patterns. The vertical scale has been multiplied by a factor of 5 for 2θ > 12.8°. The lower row of black tick marks indicates the reflection positions for the major phase and the upper row of red tick marks is for the dihydrate impurity.

Both laboratory and synchrotron patterns could be indexed (*DICVOL06*; Louër & Boultif, 2007 ▸) on a primitive monoclinic cell having *a* = 7.34705 (5), *b* = 5.43481 (4), *c* = 11.03449 (7) Å, *β* = 103.8 (6)°, and *V* = 427.740 (5) Å^3^. The systematic absences were consistent with space group *P*2_1_ (No. 4). All attempts to solve the structure using direct methods, charge flipping, and Monte Carlo simulated annealing (using a citrate and 3 Na) failed using this unit cell. Using the synchrotron pattern was complicated by the presence of 12.8 (1) wt% Na_3_(C_6_H_5_O_7_)(H_2_O)_2_ (UMOGAE; Fischer & Palladino, 2003 ▸). Since the cell of the anhydrous compound is approximately ½*a*, ½*b*, *c* that of the *C*2*/c* cell of UMOGAE, unsuccessful attempts to solve the structure were also made in 2× and 4× supercells of the observed cell. The powder pattern (Fig. 4 ▸) was indexed using *Jade 9.5* (MDI, 2012 ▸). Pseudo-Voigt profile coefficients were as parameterized in Thompson *et al.* (1987 ▸), and the asymmetry correction of Finger *et al.* (1994 ▸) was applied and microstrain broadening by Stephens (1999 ▸).

The structure was ultimately solved with *FOX* (Favre-Nicolin & Černý, 2002 ▸) using laboratory data from a single-phase dehydrated sample. A single Na_3_(C_6_H_5_O_7_) fragment was derived from UMOGAE, with Na bound to the hydroxyl group, the central carboxyl group, and one of the terminal carboxyl groups. Attempts were made using both bump-check and bond-valence restraints, but the ultimate solution came without applying these restraints. This model refined reasonably well, but the bond-valence sums of the Na atom were unreasonable. A Hartree–Fock geometry optimization was carried out using *CRYSTAL09* (Dovesi *et al.*, 2005 ▸), and the resulting model (which had Na bond-valence sums 2) led to a successful refinement. All C—C and C—O bond lengths were restrained, as were all bond angles. The hydrogen atoms were included at fixed positions, which were re-calculated using *Materials Studio* (Dassault Systemes, 2014 ▸) during the course of the refinement. The *U*_iso_ of C2, C3, and C4 were constrained to be equal, and those of H7, H8, H9, and H10 were constrained to be 1.3 × that of these carbon atoms. The *U*_iso_ of C1, C5, C6 and the oxygen atoms were constrained to be equal, and that of H18 was constrained to be 1.3 × this value. Crystal data, data collection and structure refinement details are summarized in Table 2 ▸. The structure of the UMOGAE impurity was not refined.

The Bravais–Friedel–Donnay–Harker (Bravais, 1866 ▸; Friedel, 1907 ▸; Donnay & Harker, 1937 ▸) morphology suggests that we might expect platy morphology for tris­odium citrate, with {001} as the principal faces. No texture model was necessary in the refinement, showing that preferred orientation was not significant for the rotated capillary specimen.

## DFT calculations   

After the Rietveld refinement, a density functional geometry optimization (fixed experimental unit cell) was carried out using *CRYSTAL09* (Dovesi *et al.*, 2005 ▸). The basis sets for the C, H, and O atoms were those of Gatti *et al.* (1994 ▸), and the basis set for Na was that of Dovesi *et al.* (1991 ▸). The calculation used 8 *k*-points and the B3LYP functional, and took about 42 h on a 2.8 GHz PC. The *U*_iso_ from the Rietveld refinement were assigned to the optimized fractional coordinates.

## Supplementary Material

Crystal structure: contains datablock(s) NA3CITRATE_publ, Na3Citrate_DFT, NA3CITRATE_overall, Na3Citrate_phase_1, Na3Citrate_phase_2, NA3CITRATE_p_01. DOI: 10.1107/S2056989016007453/vn2111sup1.cif

Supporting information file. DOI: 10.1107/S2056989016007453/vn2111Na3Citrate_phase_1sup2.cml

CCDC references: 1478189, 1478188, 1478190

Additional supporting information:  crystallographic information; 3D view; checkCIF report

## Figures and Tables

**Table 1:** Hydrogen-bond geometry (Å, °)

*D*—H⋯*A*	*D*—H	H⋯*A*	*D*⋯*A*	*D*—H⋯*A*
O17—H18⋯O14	0.987	1.805	2.671	144.0
C2—H8⋯O17^i^	1.086	2.356	3.355	152.2

Symmetry code: (i) 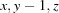.

**Table 2:** Experimental details

	Phase_1	Phase_2
Crystal data		
Chemical formula	Na_3_(C_6_H_5_O_7_)	C_6_H_5_O_7_·2H_2_O
*M*_r_	258.07	98.03
Crystal system, space group	Monoclinic, *P*2_1_	Monoclinic, *C*2/*c*
Temperature (K)	293	293
*a*, *b*, *c* (Å)	7.34705 (5), 5.43482 (4), 11.03447 (7)	15.7057 (5), 12.5045 (5), 11.2945 (8)
β (°)	103.8797 (6)	103.611 (4)
*V* (Å^3^)	427.74 (1)	2155.84 (12)
*Z*	2	2
Radiation type	Synchrotron, λ = 0.41307 Å	Synchrotron, λ = 0.41307 Å
μ (mm^−1^)	0.02	0.02
Specimen shape, size (mm)	Cylinder, 1.5 × 1.5	Cylinder, 1.5 × 1.5

Data collection		
Diffractometer	11-BM APS	11-BM APS
Specimen mounting	Kapton capillary	Kapton capillary
Data collection mode	Transmission	Transmission
Scan method	Step	Step
2θ values (°)	2θ_min_ = 0.5 2θ_max_ = 50.0 2θ_step_ = 0.001	2θ_min_ = 0.5 2θ_max_ = 50.0 2θ_step_ = 0.001

Refinement		
*R* factors and goodness of fit	*R*_p_ = 0.059, *R*_wp_ = 0.073, *R*_exp_ = 0.062, *R*(*F* ^2^) = 0.06382, χ^2^ = 1.416	*R*_p_ = 0.059, *R*_wp_ = 0.073, *R*_exp_ = 0.062, *R*(*F* ^2^) = 0.06382, χ^2^ = 1.416
No. of parameters	73	73
No. of restraints	29	29

The same symmetry and lattice parameters were used for the DFT calculation. Computer programs: *DIFFRAC* (Bruker, 2009 ▸), *PowDLL* (Kourkoumelis, 2013 ▸), *GSAS* (Larson & Von Dreele, 2004 ▸), *EXPGUI* (Toby, 2001 ▸), *DIAMOND* (Brandenburg, 2006 ▸) and *publCIF* (Westrip, 2010 ▸).

## supplementary crystallographic information

### Crystal data

**Table:**

Na_3_C_6_H_5_O_7_	β = 103.8797°
*M**_r_* = 258.07	*V* = 427.72 Å^3^
Monoclinic, *P*2_1_	*Z* = 2
*a* = 7.3471 Å	None radiation, λ = 1.5418 Å
*b* = 5.4348 Å	*T* = 300 K
*c* = 11.0345 Å	

### Data collection

**Table:**

Density functional calculation	

### Fractional atomic coordinates and isotropic or equivalent isotropic displacement parameters (Å^2^)

**Table:**

	*x*	*y*	*z*	*U*_iso_*/*U*_eq_	
C1	0.52139	0.36379	0.19408	0.01570*	
C2	0.68978	0.38591	0.30743	0.00670*	
C3	0.85441	0.53721	0.28438	0.00670*	
C4	1.03620	0.47678	0.38367	0.00670*	
C5	1.20386	0.64379	0.38297	0.01570*	
C6	0.88406	0.47193	0.15320	0.01570*	
H7	0.64280	0.46663	0.38548	0.00870*	
H8	0.74029	0.20195	0.33589	0.00870*	
H9	1.00831	0.48799	0.47670	0.00870*	
H10	1.08026	0.28777	0.37225	0.00870*	
O11	0.47255	0.54929	0.12466	0.01570*	
O12	0.44290	0.15314	0.17676	0.01570*	
O13	1.36310	0.57603	0.44517	0.01570*	
O14	1.17738	0.84623	0.32240	0.01570*	
O15	0.88375	0.24614	0.12627	0.01570*	
O16	0.90355	0.64680	0.08168	0.01570*	
O17	0.80788	0.79240	0.29212	0.01570*	
H18	0.92601	0.87924	0.29256	0.02050*	
Na19	−0.12326	0.46644	−0.11139	0.02321*	
Na20	−0.35267	−0.09861	0.07904	0.02321*	
Na21	0.50371	−0.08736	0.36215	0.02321*	

### Bond lengths (Å)

**Table:**

C1—C2	1.539	C4—C5	1.532
C1—O11	1.265	C4—H9	1.096
C1—O12	1.276	C4—H10	1.093
C2—C3	1.533	C5—O13	1.261
C2—H7	1.094	C5—O14	1.278
C2—H8	1.086	C6—O15	1.262
C3—C4	1.546	C6—O16	1.265
C3—C6	1.556	O17—H18	0.987
C3—O17	1.436		

### Hydrogen-bond geometry (Å, º)

**Table:**

*D*—H···*A*	*D*—H	H···*A*	*D*···*A*	*D*—H···*A*
O17—H18···O14	0.987	1.805	2.671	144.0
C2—H8···O17^i^	1.086	2.356	3.355	152.2

Symmetry code: (i) *x*, *y*−1, *z*.
